# A new insight into the pathway behind spontaneous recurrent pregnancy loss: decreased CYR61 gene expression

**DOI:** 10.1590/1806-9282.20231673

**Published:** 2024-07-19

**Authors:** Fahri Burcin Firatligil, Burcu Firatligil Yildirir, Ozden Yalcin-Ozuysal

**Affiliations:** 1 nkara Etlik City Hospital, Department of Obstetrics and Gynecology, Division of Perinatology – Ankara, Turkey.; 2 Tampere University, Faculty of Engineering and Natural Sciences – Tampere, Finland.; 3 Izmir Institute of Technology, Department of Molecular Biology and Genetics – Izmir, Turkey.

**Keywords:** Cytogenetics, Miscarriage, Molecular genetics

## Abstract

**OBJECTIVE:**

Investigating the potential role of CYR61 in recurrent pregnancy loss is critical for developing diagnostic approaches and treatments for recurrent pregnancy loss.

**METHODS:**

In this prospective case-control study, we have investigated the expression patterns of CYR61 in blood samples from participants with recurrent pregnancy loss in their medical history and control group (n=20 vs n=10). Peripheral blood mononuclear cells from study and control groups were isolated and the expression patterns of the CYR61 gene were determined by real-time semi-quantitative reverse transcriptase PCR.

**RESULTS:**

A significant decrease in CYR61 gene expression was demonstrated in patients with two or more clinically recognized miscarriages compared with patients without miscarriages or with a history of miscarriage (p<0.01), which may make the CYR61 gene a potential candidate for predicting the risk of recurrent pregnancy loss.

**DISCUSSION:**

This study provides a basis for a detailed investigation of candidate biomarkers and molecular players involved in the development of recurrent pregnancy loss and for the development of potential treatment approaches to prevent recurrent pregnancy loss.

## INTRODUCTION

Spontaneous abortion is the loss of a pregnancy that occurs before 20 weeks of gestation or when the fetus weighs less than 500 g^1,2^. Recurrent pregnancy loss (RPL) is the occurrence of two or more clinically established pregnancy losses within 20 weeks of conception^3^. RPL can be seen in approximately 3–5% of pregnancies^4^, which has significant negative public health implications and can be both emotionally and physically traumatizing for any couple^3^. Two types of unexplained RPLs have been identified: Type I and Type II RPL. Type I unexplained RPL is predominantly seen in women with no known underlying pathology, while Type II unexplained RPL occurs because of existing pathology that was not previously identified by routine clinical examination^5^. This type has a worse prognosis than women of similar age with unexplained type I RPL. Although the underlying cause remains unknown in approximately 50% of RPL cases, recent studies have found that most RPL cases can be associated with multiple causes, including heredity, parental age, antiphospholipid syndrome, uterine abnormalities, thrombosis, spermatogenesis, hormone metabolic or autoimmune disorders, psychological-environmental factors, and abnormalities of vascular system development and/or implantation^3,6,7^.

Vascular system development and implantation play a crucial role in embryogenesis and placental formation^8,9^. The vascular system consists of two pathways: the first is vasculogenesis, which is the formation of blood vessels in situ from angioblasts, whereas the second is angiogenesis, which is the formation of blood vessels by sprouting from pre-existing vessels^8,10^. Implantation is considered the second step in embryonic development after fertilization. In this process, the adhesion of the blastocyst to the endometrial surface is followed by its invasion through the epithelial basement membrane of the endometrium to form the placenta^11^. Therefore, any problem observed in these steps may lead to various serious problems, such as preeclampsia, intrauterine fetal demise, fetal growth restriction, spontaneous abortion, and RPL^10,11^.

CYR61 is cysteine-rich protein 61, which belongs to the CCN family and is an extracellular matrix-associated angiogenic inducer that acts as a ligand of integrins to promote cell adhesion, migration, and proliferation during placental development^8^. During embryogenesis, CYR61 is identified as one of the important factors secreted by various cell types, particularly trophoblasts, which form the outermost membrane of a blastocyst^12^. These cells play an important role in providing nutrients and regulating blood flow to the embryo, thus forming an efficient maternal–fetal vascular relationship^12^. Because CYR61 is one of the major players in trophoblast invasion during placental development, it may be a functionally important biomarker for the development of a successful pregnancy, so its downregulation may cause RPL.

In this study, we hypothesized that reduced CYR61 levels are associated with RPL. We analyzed the expression patterns of CYR61 in blood samples from participants with RPL in their medical history. Investigating the potential role of CYR61 in RPL is critical for developing diagnostic approaches and treatments for RPL.

## METHODS

This case-control study was prospectively conducted in the Perinatology Department of Etlik Zubeyde Hanim Women's Health Education and Training Hospital and the Department of Molecular Biology and Genetics of Izmir Institute of Technology between March 2019 and March 2022. This study was conducted in accordance with the principles of the Declaration of Helsinki, and the local ethics committee of Dokuz Eylul University of Izmir provided its approval (May 26, 2016; no: 2016/14-39).

### Inclusion–exclusion criteria

Screening for RPL was based on the consensus of The American College of Obstetricians and Gynecologists (ACOG)^13^ and the European Society of Human Reproduction (ESHRE)^14^.

Recurrent pregnancy loss was defined as two or more pregnancy losses before 20 weeks of gestation. The RPL group consists of RPLs with a diagnosis of missed abortion, anembryonic pregnancy, or inevitable abortion. Healthier or low-risk pregnancies (one or no abortion) were included in the control group. Pregnant women were excluded from the study if they had any of the following risk factors or conditions: uterine morphological pathologies; comorbid maternal diseases (e.g., endocrine disease, rheumatological disease, and autoimmune disorders); inflammatory conditions (presence of autoimmune disease and/or chronic inflammatory disease; the presence of infection (especially urinary tract infection, upper or lower respiratory tract infection, pelvic inflammatory disease, and active or inactive coronavirus infection, etc.); and any condition that may compromise the immune system (e.g., use of corticosteroids, antioxidants or anti-inflammatory drugs, smoking, liver and/or kidney disease, cancer, organ, or bone marrow transplant).

### Data

A total of 30 participants were included in our study: 20 of them were included in the study group, while the rest were included in the control group. The data of both groups such as demographic information (age, gravidity, and parity), anthropometric parameters of the participants [height and weight to determine body mass index (BMI)], and smoking status were obtained from the medical history of patients.

### Study design

Women with RPL who received outpatient or inpatient care and whose pregnancy ended at our hospital were included in the study group (group I) (n=20). Healthy pregnant women with low risk, who had one or no abortion in the past, and whose pregnancy ended in the same hospital were included in the control group (group II) (n=10).

After written informed consent was obtained from the participants, 5 mL of venous blood was collected from each patient with the anticoagulant ethylenediaminetetraacetic acid (EDTA) to prevent blood clotting. In the study group, venous blood samples were taken on the 20th day after the end of pregnancy or after puerperium. In the control group, venous blood samples were taken after the puerperium or on the 20th day of pregnancy. Transportation of the blood samples was in accordance with the regulations of the Department of Transportation (DOT) and the International Air Transport Association (IATA).

### Peripheral blood mononuclear cell isolation

Peripheral blood samples from 20 patients and 10 control groups were used in the study. Mononuclear cells from human blood samples were isolated using density gradient centrifugation and Ficoll-Paque solution. Blood samples were diluted with 2–4 times the volume of cold 1× PBS buffer and mixed well by pipetting up and down. The diluted blood suspension was then carefully layered over 10–15 mL of Ficoll-Paque in a 50-mL Falcon tube and centrifuged at 400×g for 40 min at RT without brake. Later, the upper layer was aspirated leaving the cell layer undisturbed at the interphase. The mononuclear cell layer was then carefully transferred to a new 50-mL Falcon tube, and the tube was filled with cold 1× PBS buffer, mixed, and centrifuged at 300×g for 10 min at RT. After the supernatant was carefully removed completely, the cell pellet was resuspended with 1× PBS and centrifuged at 200×g for 10 min at RT. This step was repeated twice, and the cell pellet was resuspended with lysis buffer containing beta-mercaptoethanol to proceed with RNA isolation.

### RNA isolation and real-time semi-quantitative reverse transcriptase PCR

RNA isolation was performed according to the protocol of the Pure Link RNA Mini Kit. cDNAs were synthesized from 1 μg total RNAs using the Revert Aid First Strand cDNA Synthesis Kit (K1622, Thermo-Fisher Scientific, USA). The mRNA levels were analyzed by real-time semi-quantitative reverse transcriptase PCR (RT-qPCR) using the Fast Start Essential DNA Green Master Kit (06402712001, Roche) on the Light Cycler® 96 instrument. Relative expression levels of the CYR61 gene were calculated by the Delta-Ct method using human TATA box binding protein (TBP) as the housekeeping gene. Non-template controls were also included in each condition.

### Statistical analysis

The demographic and clinical characteristics of patients were summarized with means, standard deviations, and median values to provide a description of the patients. Gene expression values and differences in demographic and clinical characteristics between patient groups were analyzed using a two-tailed t-test, and a p<0.05 was considered statistically significant.

## RESULTS

The demographic and clinical characteristics of the study groups (Groups I and II) are shown in Table 1. The effects of age, gravidity and parity, and body mass index on the development of RPL were examined. No data were available on the smoking status of the patients. There were no significant differences between the groups in terms of age, number of pregnancies and births, and BMI for the development of RPL in the patients.

**Table 1:** Demographic and clinical characteristics of the studied groups.

	Group I (mean±SD)	Group II (mean±SD)	p-value
Age (years)	29.25±4.56	32±3.53	0.9738
Gravidity (numbers)	3.58±1.55	3±0.70	0.875
Parity (numbers)	1.08±1.18	3.25±1.08	0.189
Abortion (numbers)	2.5±0.86	0.75±0.43	0.0195
BMI (kg/m^2^)	24.9±4.64	28.4±2.67	0.266
Smoking status	0	0	NA

BMI: body mass index; SD: standard deviation; p<0.05.

**Figure 1:**
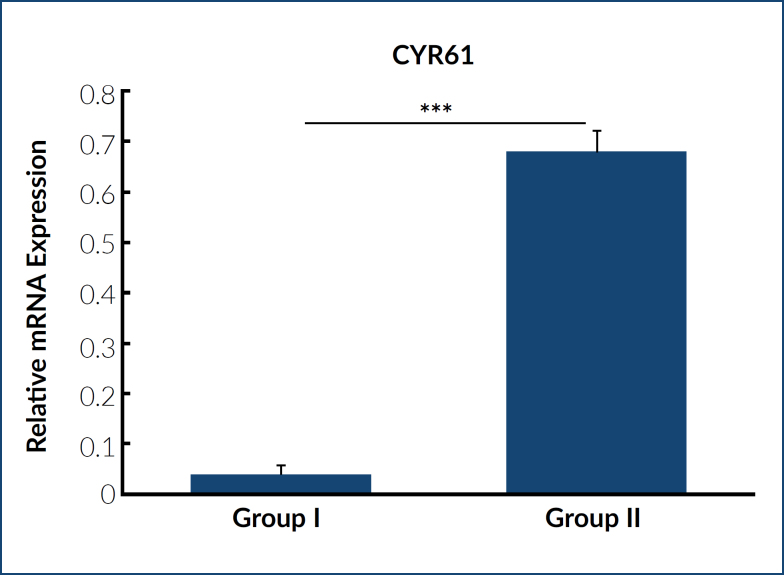
The expression profile of CYR61 in patients in both groups. The relative mRNA expression levels of the CYR61 gene are shown (experiments were performed in triplicate and repeated thrice). ***p<0.01.

We hypothesized that CYR61 expression levels might be lower in patients with miscarriages compared with patients without miscarriages or one miscarriage history. This hypothesis is based on the observation that decreased expression of the CYR61 gene in mice leads to embryonic lethality because of impaired vascular integrity in embryonic arteries^15^. Therefore, we examined CYR61 expression levels in both groups of patients. As expected, CYR61 expression was significantly lower in patients with a history of RPL in their previous pregnancies than in patients who had one or no miscarriage (Figure 1).

## DISCUSSION

Recurrent pregnancy loss is the condition in which there are two or more clinically recognized miscarriages before the gestation of 20 weeks. RPL is a complex and usually ill-defined situation in reproductive medicine. The etiology underlying approximately 50% of RPL patients remains unclear, causing frustration and concern for patients and their families.

Zhang et al.^16^ attempted to uncover the molecular mechanism of preeclampsia caused by miR-155. Their findings provide new evidence that abnormally expressed miR-155 may play an important role in the development of preeclampsia through the downregulation of CYR61^16^. CYR61 is highly expressed in the human placenta^16^. It is mainly expressed in endothelial cells, villous stromal cells, and interstitial extravillous trophoblast giant cells and is involved in angiogenic processes and in the migratory properties of extravillous trophoblast cells in the developing human placenta^8,15^. In severe preeclampsia, Zhang et al.^16^ showed that pregnant women have reduced expression of the CYR61 gene.

CYR61 gene expression in the context of RPL has not yet been investigated in English-language medical research. In this study, we wanted to show the relationship between downregulation of the CYR61 gene and RPL. Our results showed that a significant downregulation of CYR61 expression was observed in patients with a history of RPL, indicating the importance of CYR61 for the development of a successful pregnancy. Therefore, the CYR61 gene can be considered one of the most important biomarkers for the assessment of RPL risk in human patients, especially in those who have a history of abortion. However, the causative role or functional significance of the CYR61 gene should be investigated with a view to developing new treatment and/or prevention approaches. Although the expression levels obtained from the patients' PBMCs give an idea of the importance of the CYR61 gene in a successful pregnancy, the tissue-specific expression of CYR61 should be examined to gain a deeper understanding of its role in pregnancy and RPL.

### Ethics

All patients and controls provided written informed consents, and this study was approved by the Committee on Ethics of Dokuz Eylul University, Faculty of Medicine, Izmir, Turkey.
